# New insights into clade 1 influenza A (H5N1) virus circulation in Cambodia and within the Southern Indochina peninsula

**Published:** 2011-01-10

**Authors:** San Sorn, Sek Mardy, Davun Holl, Sirenda Vong, Philippe Buchy

**Affiliations:** 1 Institut Pasteur in Cambodia, Phnom Penh, Cambodia; 2 National Veterinary Research Institute, Ministry of Agriculture, Phnom Penh, Cambodia

## Background

In Cambodia, the highly pathogenic avian influenza (HPAI) A subtype H5N1 virus was detected for the first time in January 2004. From 2004 to 2010, there have been 26 outbreaks in poultry and 10 human cases reported in the country but the origin of these epizootics remains unclear.

## Methods

The phylogenetic relationships among the H5N1 strains were reconstructed by neighbour-joining and Bayesians methods. We analyzed the sequences of all 8 genomic segments of 40 H5N1 Cambodian viruses together with sequences from over 100 isolates from Southeast Asia including Vietnam, Thailand and Laos.

## Results

All viruses isolated in Cambodia since 2004 belong to clade 1, genotype Z. Based on phylogenetic relationships, HPAI H5N1 virus was probably introduced from Thailand in 2004. In 2005 and 2006, several sublineages emerged in Cambodia and were probably the result of multiple introductions of H5N1 virus from Vietnam where similar strains were detected before outbreaks occurred in Cambodia. Interestingly, in 2006, we observed a north to south spread of the virus following a main road. A new sublineage appeared in summer 2006. Since then, all viruses isolated in Cambodia and South Vietnam clustered into this group, suggesting that this sublineage became endemic in the Southern Indochina peninsula. Other clades which have been imported to neighboring countries by migratory birds (i.e., clade 2.2) have not been detected in Cambodia.

## Conclusion

Cambodia is essentially a poultry-importing country. The first poultry deaths were observed in semi-industrial farms that imported broiler and layer parental stocks from a sister company in Thailand, where concomitant outbreaks occurred. Then, multiple introductions of H5N1 viruses most likely occurred through illegal trading in poultry from Vietnam. Our data suggest that the clade 1 H5N1 virus is spreading essentially through poultry trading, particularly along road transportation routes. The role of the migratory birds appeared to be at most limited to local or regional transmission. The mechanisms described here would explain the maintenance and extension of the H5N1 virus within the Cambodia-South Vietnam regions for the last 6 years. This also highlights the persistent risk of H5N1 virus transmission in humans in the region while the new H1N1 pandemic virus that affects millions of humans is also frequently detected in pigs and shows a dangerous propensity to recombine.

